# Multi-target pharmacology: possibilities and limitations of the “skeleton key approach” from a medicinal chemist perspective

**DOI:** 10.3389/fphar.2015.00205

**Published:** 2015-09-22

**Authors:** Alan Talevi

**Affiliations:** Medicinal Chemistry, Department of Biological Sciences, Faculty of Exact Sciences, National University of La Plata, La Plata, Argentina

**Keywords:** multi-target agents, lock and key paradigm, gene profile, drug resistance, drug repositioning, drug design, designed multiple ligands

## Abstract

Multi-target drugs have raised considerable interest in the last decade owing to their advantages in the treatment of complex diseases and health conditions linked to drug resistance issues. Prospective drug repositioning to treat comorbid conditions is an additional, overlooked application of multi-target ligands. While medicinal chemists usually rely on some version of the lock and key paradigm to design novel therapeutics, modern pharmacology recognizes that the mid- and long-term effects of a given drug on a biological system may depend not only on the specific ligand-target recognition events but also on the influence of the repeated administration of a drug on the cell gene signature. The design of multi-target agents usually imposes challenging restrictions on the topology or flexibility of the candidate drugs, which are briefly discussed in the present article. Finally, computational strategies to approach the identification of novel multi-target agents are overviewed.

## Introduction

Multi-target drugs (or multi-functional drugs or network therapeutics) have attracted considerable attention in the last decade, as potential therapeutic solutions to diseases of complex etiology (Talevi et al., 2012; Koerberle and Werz, 2014; Zheng et al., 2014) and health conditions linked to drug-resistance issues (Talevi and Bruno-Blanch, 2013; Li et al., 2014). According to the “one drug, one target” paradigm, highly potent and specific (single-target) treatments would be better tolerated due to absence of off-target side-effects. However, poor correlation between *in vitro* drug effects and *in vivo* efficacy is often found with target-driven approximations (Kell, 2013; Margineanu, 2014). While target-first strategies might prove useful to approach single gene disorders, disease is often a multifactorial condition involving a combination of constitutive and/or environmental factors. Owing to compensatory mechanisms and redundant functions, biological systems are resilient to single-point perturbations (Hopkins, 2008). Under such perspective, disease often results from the breakdown of robust physiological systems due to multiple genetic and/or environmental factors, leading to the establishment of robust disease conditions (Yildrim et al., 2007). Thus, complex disorders are more likely to be healed or alleviated though simultaneous modulation of multiple targets.

Though this strategy has only been purposely applied in the last 10 to 15 years, many of the previously known therapeutic agents are in fact multi-target ligands (Yildrim et al., 2007), which is especially true for those drugs that were discovered by serendipity, phenotypic screening or traditional medicine. Note that in all these cases, the knowledge on the pharmacological effect precedes the knowledge of the mode of action. Aspirin itself has been shown to act through a diversity of molecular mechanisms besides cyclooxygenase inhibition (Koerberle and Werz, 2014). Some therapeutic categories, e.g., mood disorder medications, are particularly abundant on classical examples of multi-target drugs (Roth et al., 2004). So actually, multi-target drugs have long been known and effectively used in the clinical practice but have majorly been found serendipitously or through phenotypic screening. What are the possibilities and limitations of tailored multi-target drugs?

## Revisiting and Squeezing the Classical Lock and Key Paradigm

**FIGURE 1:**
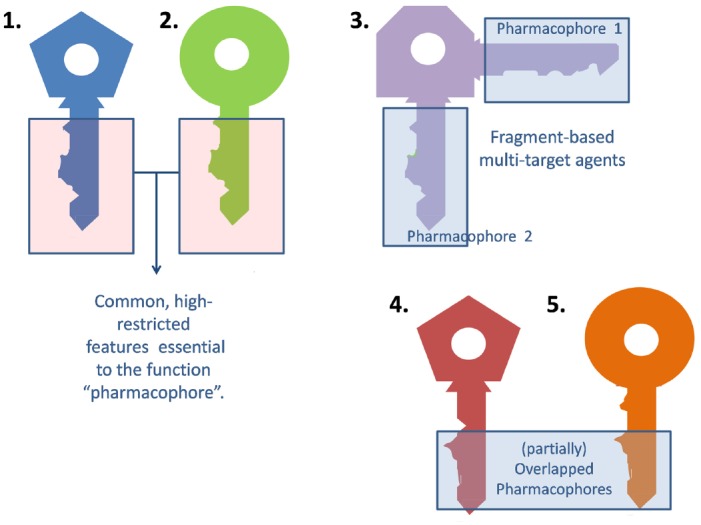
**Extrapolation of the classic lock and key analogy to multi-target agents**.

Medicinal chemists usually resort to the traditional lock and key model to describe the interaction between a ligand and its molecular target (or an updated version of this paradigm that contemplates the ligand and target flexibility, such as the hand-in-glove analogy). The general idea is that the ligand (*the key*) and the target (*the lock*) should have complementary features to efficiently interact and trigger some biological response (*open the lock*). Frequently, different ligands can elicit a qualitatively similar response at a certain target. For different keys to activate the same lock alike they must share some common, essential arrangement of features (*the blade of the key*), which will be termed the *pharmacophore* (from the Greek, *what carries the medicine*). The remaining part of the key (*the bow*) may be indeed important, but less subject to structural restrictions (Figure 1).

A multi-target ligand might be conceived as a *skeleton or master key* capable of unlocking several locks. While selective non-selectivity might be of benefit, promiscuity (non-selective non-selectivity) might in contrast raise severe safety concerns and should be avoided. Why may a non-promiscuous ligand activate different targets? There are many possible answers to this question. First, it is frequent for a given ligand to act on several isoforms of the same protein. For instance, xilocaine (lidocaine) can produce anesthetic, antiarrhythmic and anticonvulsant effects by blocking the peripheral nervous system, heart and central nervous system sodium channels (Catterall, 2000). Alternatively, different members of a given biochemical pathway might share, to some extent, ligand specificity due to co-evolution. Finally, a ligand might display affinity to two or more unrelated targets by combining different pharmacophores in the same molecule (Morphy et al., 2004). Frequently, such combination of pharmacophores leads to molecules that are either enthalpically or entropically unfavorable, which conspires against the design of multi-target drugs, as will be later discussed in the correspondent section. This is metaphorically represented in Figure 1, through the awkward design of key number 3.

The contribution of biotechnology, however, has made very clear that the lock and key analogy can fall short to explain the effects of a drug on a biological system, particularly when medium- and long-term drug exposure (multiple-dose regimens) is required. After sustained exposure to a chemical agent the gene signature of a cell varies: some genes are upregulated while others are downregulated (e.g., owing to activation of nuclear receptors, compensatory mechanisms, etc.). Whereas in the past attention was directed to the direct interactions between the drug and its molecular target/s, now it is known that a more holistic perspective is needed to fully characterize the action of a drug on a biological system. For example, it has been reported that chronic administration of valproic acid and carbamazepine downregulates cytosolic phospholipase A2 and/or cyclooxygenase (with the consequent reduction of proinflammatory cytokines; Bosetti et al., 2003; Gherlardoni et al., 2004), an effect that may be involved in the effectiveness of these agents in epilepsy and bipolar disorder. The need for such holistic view is unequivocally expressed in the Connectivity Map, a publicly available resource meant to connect disease and small molecules through gene-profiles (Qu and Rajpal, 2012). The Connectivity Map stores gene expression profiles derived from the treatment of human cells cultured with a large number of drugs; when a disease signature is used as a query, it is expected that those drugs related to the disease by opposite expression changes (inverse similarity) will be potential treatments.

## Possible Applications of Multi-Target Ligands

Three main applications of multi-target agents in a therapeutic can be envisioned.

### Complex Disorders

Complex disorders are multi-factorial health conditions triggered by a number of intrinsic and/or environmental factors acting together on an organism. Among them we may mention mood disorders, neurodegenerative diseases, chronic inflammation or cancer. Despite the advances on the comprehension of the biological basis of these conditions and the huge investments made by the pharmaceutical sector, pharmaceutical solutions remain elusive. Although in some cases such disorders can be or are approached through combined therapies, multi-target ligands would present clear advantages, among them more predictive pharmacokinetics, better patient compliance, and reduced risk of drug interactions. There are several reviews available covering the potential of the multi-target approach in cancer (Petrelli and Giordano, 2008; Petrelli and Valabrega, 2009), Alzheimer’s disease (Bajda et al., 2011; Dias and Viegas, 2014; Zheng et al., 2014), Parkinson’s disease (Youdim et al., 2014), inflammation (Hwang et al., 2013), depression and other psychiatric disorders (Wong et al., 2008; Milan, 2014).

### Drug Resistance

Simultaneously impacting different targets could also be advantageous to approach individuals expressing intrinsic or induced variability in drug response due to modifications in key disease-relevant biological pathways and activation of compensatory mechanisms (Zimmermann et al., 2007; Xie et al., 2012). Apart from the obvious applications in the field of antimicrobial chemotherapy (it is less probable to develop resistance linked to single-point mutations against multi-target than single-target agents) this strategy could also be pertinent to treat non-infectious conditions characterized by high incidence of the drug resistance phenomena, e.g., epilepsy (Bianchi et al., 2009; Margineanu, 2014). One third of the epileptic patients suffer from refractory epilepsy. One of the prevalent hypotheses to explain refractory epilepsy cases proposes that at least part of the non-responsive patients might express variations in molecular targets of antiepileptic drugs (Talevi and Bruno-Blanch, 2013). Isobolographic studies in animal models and clinical experience suggest that combination of drugs with different mechanisms tends to be beneficial (Kwan and Brodie, 2006; Kaminski et al., 2009; Lee and Dworetzky, 2010; Brodie et al., 2011). On the other hand, while there exists consensus regarding the utility of single-target drugs for the treatment of some specific epilepsy types or syndromes, broad spectrum antiepileptic drugs such as valproic acid are among the most used antiepileptic agents and might be valuable in those cases where, at the onset of epilepsy, diagnosis of the specific syndrome is elusive (Bourgeois, 2007; Lagae, 2009; Löscher et al., 2013; Margineanu, 2014).

### Prospective Drug Repositioning

Drug repositioning (i.e., finding a second or further medical use for already known therapeutics, including approved, discontinued, shelved, and experimental drugs) has attracted enormous interest within the academic and pharmaceutical sectors during the last 10 years (Ashburn and Thor, 2004; Novac, 2013). Most of the successful drug repositioning cases have been found by serendipity or through exploitation of the original action mechanism of a drug for new indications (*on target* repositioning). Multi-target agents are natural candidates for more innovative, *off-target* drug repositioning. Computational approaches to drug repositioning have so far focused on what we will call *retrospective drug repositioning*: screening known drugs collections/libraries to find novel indications for already known therapeutic agents. *Prospective drug repositioning*, in contrast, would explore drug repositioning possibilities much earlier in the drug discovery process. While some pharmaceutical companies now consider exploring repositioning alternatives for drugs in the pipeline, the approach could be taken much further, by designing multi-purpose drugs to treat different conditions; prominently, frequently co-morbid disorders (e.g., diabetes and cardiac disease; anxiety and peptic ulcer disease, epilepsy, and depression) or, alternatively, underlying pathologies plus disease symptoms. The case of amiodarone and related compounds and Chagas disease can be illustrative. Chagas disease is a tropical parasitic disease historically endemic to Latin America. The late phase of the disease is characterized by life-threatening heart disorder in around one third of the patients. Amiodarone is a class III antiarrhythmic agent that shares many characteristics of other electrophysiological anti-arrhythmic drugs, including inhibition of sodium and potassium channels and L-type calcium channels. Interestingly, some studies showed that patients with chagasic cardiomyopathy treated with amiodarone had a more rapid recovery when compared with other patients treated with class I and class IV antiarrhythmics. This fact suggested that other mode of action could be in play. It was later demonstrated that amiodarone was able to act directly on the parasite survival, affecting the growth of *Trypanosoma cruzi* extracellular epimastigotes and *T. cruzi* amastigotes (that is, amiodarone could act on the underlying pathology). The mechanism of action of the drug was elucidated, showing that this drug directly disrupts the intracellular calcium regulation of the parasite (Benaim et al., 2006). Similar results were later observed with dronedarone (Benaim et al., 2012). Still, this example is another case of retrospective drug repositioning, since the new medical use emerged from clinical observations. A future challenge is to define whether this kind of indication expansion oriented to the treatment of co-morbid conditions could be anticipated through rational approaches at early stages of the drug development process, thus helping to provide evidence on possible advantages of new treatments compared to the existent ones, and additional criteria to decide which drug candidates should be prioritized to clinical trials and to conveniently choose the clinical endpoints of the trial that will be used to test superiority or non-superiority of the treatments under comparison. Computational network-based approximations could prove valuable to unveil hidden connections between diseases and assist these types of initiatives.

## Some Considerations Related to the Design and Screening of Multi-Target Agents

**FIGURE 2:**
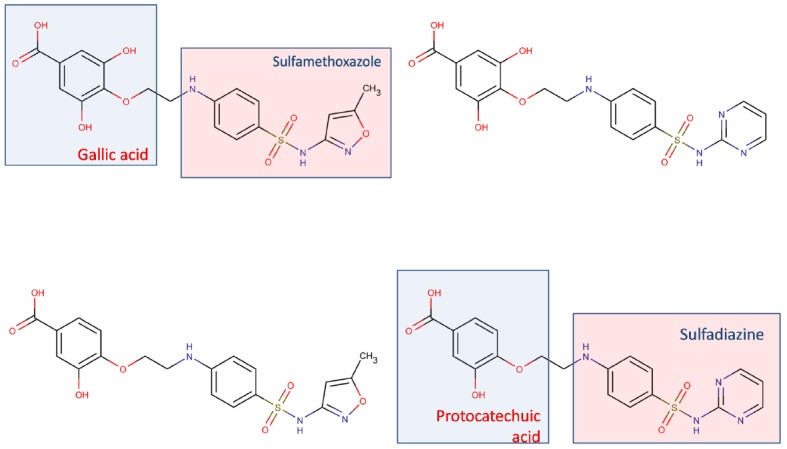
**Multi-target antibiotic phyto-drug conjugates designed by Jayaraman et al. (2013)**.

Development of tailored multi-target agents with affinity to unrelated or weakly related drug targets relies mainly in two approaches (Morphy et al., 2004; Ma et al., 2010): the methodical combination of pharmacophores from selective, single-target ligands (a fragment-based approach) and; the screening of compound collections by simultaneous application of multiple computational models (or a single, multi-tasking computational model) to identify compounds with a suitable combination of activities. In the first approximation, the distinct pharmacophores are joined together by a cleavable or stable linker or, alternatively, they are overlapped by taking advantage of structural commonalities (Morphy et al., 2004). The use of linkers often leads to compounds with unfavorable biopharmaceutic or pharmacokinetic profile (e.g., compounds that violate more than two of the Lipinski’s rules). Although the use of cleavable linkers might be advantageous, it also limits some of the merits of the multi-target approach in comparison with combination therapies (simplified pharmacokinetics, reduced chance of drug interactions). Moreover, the fragment-based approach could lead to poor ligand efficiency metrics (Hopkins et al., 2014), which refer to the binding efficiency *per atom*. It might be speculated that, since only a part of the molecule can interact with each of the proposed targets, the other part can become an obstacle for the binding event, reducing the binding efficiency because of enthalpic and/or entropic reasons, which is represented through the awkward topology of key number 3 (Figure 1). Therefore, the overlapping or merging approach (searching partially or highly integrated pharmacophores in a small molecule) seems more attractive from a biopharmaceutical viewpoint. Including some degree of flexibility in the molecule may help the common and non-common pharmacophoric features to accommodate to the correspondent binding sites of the different intended targets; however, the degree of flexibility should be carefully tuned so that an excess of flexibility does not conspire against the binding affinity (owing to unfavorable entropic loss associated to the binding event) or the bioavailability of the drug (it should be remembered that many druglikeness rules limit the number of flexible bonds in the molecule). An illustrative example of some of these principles is provided by the recent research from Jayaraman et al. (2013). These authors applied the fragment-based approach in the design of phytochemical-antibiotic conjugates conceived as multivalent inhibitors of *Pseudomonas aeruginosa* DNA gyrase subunit B (GyrB)/topoisomerase IV subunit B, dihydrofolate reductase (DHFR) and dihydropteroate synthase (DHPS). Departing from previously identified pharmacophores for inhibitors of *E. coli* GyrB and DHFR, the authors derived a common pharmacophoric model for multi-inhibition of such enzymes. Remarkably, they decided on using simple phenols (gallic acid and protocatechuic acid, simpler structural analogs of the bivalent natural product epigallocatechin gallate) conjugated through a non-cleavable linker to sulfamethoxazole and sulfadiazine (which inhibit DHPS; Figure 2). The decision of using simple phytochemicals as departure points resulted in four drug-like compounds with acceptable computed biopharmaceutical properties, which was checked through different drug-likeness rules (Lipinski and Veber rules) and by predicting the solubility and the percentage of absorption for the designed drug candidates. Two of the candidates displayed no violation of the rule of five and Veber rules, while the remaining two showed only one violation of Lipinski rules and marginal violation of Veber rules.

Regarding the screening approximation, one should bear in mind that the hit rate in the screening campaign is expected to be lower than the ones obtained when looking for single-target drug candidates (Talevi et al., 2012): each model used in the *in silico* screening process functions as a structural restriction that filters out all the molecules that do not gather the model requisites; thus, the more models used, the less probable it is to find chemical compounds accomplishing all the models structural constraints. For example, Nair et al. (2013) have recently performed a virtual screening campaign to identify multi-target inhibitors of DAP-kinases (a family of pro-apoptotic proteins also involve in autophagy, which are proposed as a promising target for therapeutic intervention of brain ischemia and neurodegenerative diseases). Among DAP-kinases, DRP1 has been reported to be the upstream protein of all the DAP-kinases as it is involved in the activation of other members of the family. However, modulation of DRP1 is not enough to attenuate the cell death pathways activated by DAP-kinases, owing to the existence of alternative activating sources. Searching for multi-target agents, the authors have explored a combined database of 391 known ligands of one of three members of the DAP-kinases family: DAPk1, DRP1, and ZIPk. This library was compiled from the Protein Data Bank and ChEMBL, and it was sequentially screened through three pharmacophore hypothesis of DAPk1, DRP1, and ZIPk, in that order. Screening using the first hypothesis (DAPk1) resulted in 196 hits. Further, screening of these hits by DRP1 pharmacophore resulted in 56 hits, which contained pharmacophore features of both DAPk1 and DRP1 ligands. The 56 ligand hits were then screened by the ZIPk pharmacophore, retrieving only four ligands gathering the pharmacophoric features of all three DAP-kinases. The limited number of hits obtained when using sequential *in silico* filters/models to select multi-target agents might be compensated by the huge, ever expanding available chemical universe. Multitasking QSAR approximations (Zanni et al., 2014; Speck-Planche and Cordeiro, 2015) could prove as a valuable tool to implement this strategy.

## Target Selection

So far the advantages and challenges posed by the multi-target approach have been discussed. A critical question remains, however, to be made: if we are to design multi-target agents, how shall we choose our molecular targets? Obviously, a drug target needs to have the potential to be disease modifying. Secondly, if we are fighting against an infection or a deregulated cell (e.g., in cancer) the drug must display some degree of selectivity, e.g., the drug target must be exclusively or preferentially expressed in the infectious agent or in the cancerous cell, targeted proteins in a pathogen should not have homologous proteins in the host or homologous proteins in the host should be sufficiently different from those in the pathogen, etc. Furthermore, the Medicinal Chemistry community has long accepted that not all the proteins are equally “druggable,” i.e., likely to be moderated by small molecules. A number of approaches to assess druggability have been proposed in the specialized literature, from “guilt by association” approximations (a protein is predicted to be druggable if it belongs to a protein family for which at least one member of the family is targeted by a drug) to methods based on binding site prediction, among others (Keller et al., 2006; Cheng et al., 2007). But still the previous are just general considerations valid for both single- and multi-target approximations. When aiming at multiple targets, the choice of the targets and the pursued type of inhibition depend on several factors, among them the nature of the disease (infectious disease? complex disorder?) and/or the possible mechanisms of drug resistance (adaptive mechanisms? target amplification or mutation?). A relevant issue that deserves attention is whether it is preferable to directly block the selected targets or to modulate them (e.g., through weak partial inhibitions). Under our modern paradigm, built on a systems biology perspective, it is understood that, in general, we are not targeting isolated proteins but pathways instead. We might target different signaling pathways (parallel targeting), which may be valuable to block escape routes, adaptive resistance mechanism and compensatory homeostatic responses; alternatively, vertical targeting (attacking the same pathway at different nodes) might prove useful against other types of resistance (e.g., target mutations; Shahbazian et al., 2012). When trying to kill pathogens or malignant cells, attacking hubs (highly connected nodes in a biochemical network) might be the strategy of choice; on the other hand, if the treatment objective is to restore a perturbed network to a healthy state, using low affinity multi-target ligands to modulate multiple non-crucial nodes neighboring key nodes ligands might be advantageous in order to avoid sever side-effects (that might be otherwise expected if blocking a key node with a crucial physiological function; Csernely et al., 2013). Metabolic control analysis constitutes a useful frame to evaluate the importance and relative contribution of individual metabolic steps in the overall functioning of a particular system and, subsequently, to identify optimal targets (Hornberg et al., 2007).

## Conclusion

Multi-target agents are a promising strategy to face complex, multifactor disorders and drug resistance issues. Additionally, they can prove valuable in prospective drug repositioning oriented to the treatment of comorbid conditions or both the underlying pathology and its symptoms, an overlooked application to the moment. Compared to combination therapies, they present several advantages, including more predictable pharmacokinetics, lower probabilities of drug interactions and higher patient compliance.

We have highlighted some difficulties related to the search of tailored multi-target drugs (e.g., enthalpic and entropic considerations and potential bioavailability issues, limited number of hits when sequentially screening a virtual library). Besides the classical key and lock paradigm to approach the multi-target strategy, the effect of the drug on cell gene signatures should also be considered, especially when looking for middle- and long-term treatments, which is often the case for complex disorders. Finally, network analysis might provide clues to help target selection, which is highly dependent on the nature of the treated disorder and the known mechanisms of resistance.

### Conflict of Interest Statement

The author declares that the research was conducted in the absence of any commercial or financial relationships that could be construed as a potential conflict of interest.
